# Microbiome Analysis Reveals the Presence of *Bartonella* spp. and *Acinetobacter* spp. in Deer Keds (*Lipoptena cervi*)

**DOI:** 10.3389/fmicb.2018.03100

**Published:** 2018-12-20

**Authors:** Yvonne Regier, Kassandra Komma, Markus Weigel, Arto T. Pulliainen, Stephan Göttig, Torsten Hain, Volkhard A. J. Kempf

**Affiliations:** ^1^Institute for Medical Microbiology and Infection Control, University Hospital, Goethe-University, Frankfurt am Main, Germany; ^2^Institute of Medical Microbiology, Justus-Liebig University, Giessen, Germany; ^3^Research Center for Cancer, Infections and Immunity, Institute of Biomedicine, University of Turku, Turku, Finland; ^4^German Centre for Infection Research (DZIF), Partner Site Giessen-Marburg-Langen, Giessen, Germany

**Keywords:** next generation sequencing (NGS), one health, epidemiology, wild animals, humans

## Abstract

The deer ked (*Lipoptena cervi*) is distributed in Europe, North America, and Siberia and mainly infests cervids as roe deer, fallow deer, and moose. From a one health perspective, deer keds occasionally bite other animals or humans and are a potential vector for *Bartonella schoenbuchensis*. This bacterium belongs to a lineage of ruminant-associated *Bartonella* spp. and is suspected to cause dermatitis and febrile diseases in humans. In this study, we analyzed the microbiome from 130 deer keds collected from roe deer, fallow deer and humans in the federal states of Hesse, Baden-Wuerttemberg, and Brandenburg, Germany. Endosymbiontic *Arsenophonus* spp. and *Bartonella* spp. represented the biggest portion (~90%) of the microbiome. Most *Bartonella* spp. (*n* = 93) were confirmed to represent *B. schoenbuchensis*. In deer keds collected from humans, no *Bartonella* spp. were detected. Furthermore, *Acinetobacter* spp. were present in four samples, one of those was confirmed to represent *A. baumannii*. These data suggest that deer keds harbor only a very narrow spectrum of bacteria which are potentially pathogenic for animals of humans.

## Introduction

**Figure 1:**
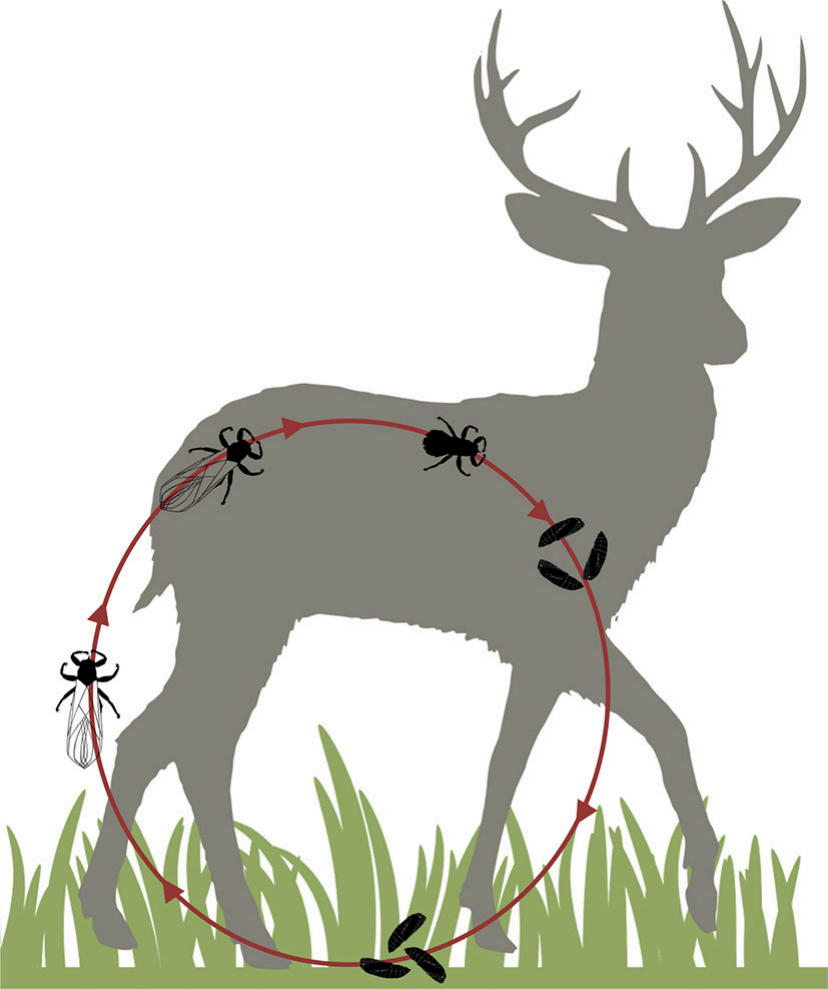
Life cycle of deer keds. The winged adults fly to suitable hosts (mainly cervids) and lose their wings before sucking blood. They give birth to living larvae which fall to the ground as pupae and remain there until new adult deer keds hatch to find new hosts.

Blood-sucking arthropods are vectors for numerous infectious agents in humans and animals and therefore of high interest in one *health* approaches. Deer keds (*Lipoptena cervi*) belong to the family of the louse flies (*Hippoboscide*) which are found in Europe, North America, and Siberia (Lindener, 1964). From August to November, the winged *imagines* (adults) fly to suitable hosts (mainly cervids) and lose their wings before they start to suck blood. They give birth to living larvae which fall to the ground as pupae and remain there until new *imagines* hatch to find new hosts (Haarløv and Haarlov, 1964). Figure 1 shows a scheme of the life cycle of deer keds.

It is unclear if the bites cause harm to the infested cervids: moose, which usually are highly infested with deer keds (Madslien et al., 2012), do not show worse indices of health compared to moose which live in deer ked-free areas (Paakkonen et al., 2012). However, hair loss in moose occurs when they are heavily infested with deer keds (Madslien et al., 2011).

Occasionally, deer keds also bite humans who can develop dermatitis (Rantanen et al., 1982) possibly caused by *Bartonella schoenbuchensis* (Dehio et al., 2004). The vector-competence of *L. cervi* for *B. schoenbuchensis* seems to be proven and is suspected for other deer ked species (*L. mazamae*) (Dehio et al., 2004; Reeves et al., 2006; Matsumoto et al., 2008; Duodu et al., 2013; Bruin et al., 2015; Korhonen et al., 2015; Szewczyk et al., 2017).

*Bartonella* spp. are Gram-negative, facultative intracellular bacteria which are usually transmitted by blood sucking arthropods and which can cause intraerythrocytic infections in their reservoir hosts (Dehio, 2005; Mändle et al., 2005; Maggi et al., 2011). The most frequently detected pathogen among in humans is *B. henselae*, the agent of cat-scratch disease. Cats are the reservoir hosts for these bacteria and pathogens are transmitted to humans by scratches or bites. Dogs can also become infected and develop endocarditis, fever of unknown origin and peliosis hepatis (Kitchell et al., 2000; Fenimore et al., 2011; Maggi et al., 2011; Drut et al., 2014). At least 37 *Bartonella* spp. are described which can infect a broad variety of mammals (Regier and Kempf, 2017). *B. schoenbuchensis* was first isolated from the blood of wild roe deer in 1999 (Dehio et al., 2001). It belongs to a lineage of ruminant-associated *Bartonella* spp. comprising of *B. schoenbuchensis, B. capreoli, B. chomelii, B. bovis*, and *B. melophagi* (Engel et al., 2011). Reports of diseases associated with these bacteria are rare for animals and humans as chronic asymptomatic infections with long lasting bacteremia are common for *Bartonella* spp. in their respective reservoir hosts (Rolain et al., 2001; Dehio et al., 2004). There is also evidence that these *Bartonella* spp. might cause endocarditis, fatigue, muscle pain and fever in their reservoir hosts or even in humans (Maillard et al., 2007; Maggi et al., 2009; Vayssier-Taussat et al., 2016). In this study we report a comprehensive microbiome analysis using next generation sequencing (NGS) to address further pathogenic agents in deer keds from Germany.

## Materials and Methods

### Sample Collection

The samples were collected between May and December 2017 from hunted roe and fallow deer at several locations in the federal state of Hesse, Germany, and nearby Karlsruhe in the federal state of Baden-Wuerttemberg, Germany. Deer keds were also sent from Ettlingen, Baden-Wuerttemberg, Germany and Wittstock, Brandenburg, Germany. Keds were collected in sterile, DNA-free tubes (Eppendorf, Hamburg, Germany) containing 70% ethanol. Whenever possible, muscle samples or full blood was collected from the hunted animals. No experimental procedures on animals or humans were performed. Only deer keds from hunted animals and from humans who sent them to us for diagnostic reasons were screened. For these procedures, there is no need for a permission from an ethics committee in Germany.

### DNA-Extraction From Deer Keds, Muscle, and Full Blood

DNA extraction from deer keds and muscle samples of hunted deer was conducted as previously described (Regier et al., 2017). All deer keds were individually removed from the tubes with sterile forceps. Each deer ked was treated individually to prevent cross- contamination rinsed once in ethanol and twice in sterile water. DNA was extracted with the QIAamp DNA Mini Kit (Qiagen, Hilden, Germany) according to manufacturer's instructions. Grinding of deer keds and muscle samples was conducted with disposable, sterile mortars and pestles. DNA from full-blood was extracted using the DNeasy Blood and Tissue Kit (Qiagen) according to manufacturer's instructions.

The laboratories of the Institute for Medical Microbiology and Infection Control at the University Hospital of the Goethe University in Frankfurt (Germany) undergo a strict quality control management (DIN ISO 15189:2014 certificate, valid through January 2021). There was no increase of *Bartonella* or *Acinetobacter* positive cases during this study; therefore, the possibility of DNA contamination from non-study material is highly unlikely.

### Microbiome Analysis of Deer Keds Using Next Generation Sequencing by Illumina Technology

The 16S rRNA gene of each deer ked DNA sample was amplified with primers for the V4 region (Caporaso et al., 2011) and analyzed as previously described (Regier et al., 2018). In brief, the V4 region amplification was done using Platinum SuperFi PCR Master Mix (Thermo Fisher Scientifc, Carlsbad, U.S.A.). Thermocycler conditions were used as described previously (Regier et al., 2018). PCR products were purified using AMPure XP DNA beads (Beckman Coulter, Brea, U.S.A.) before running the index and adapter ligation PCR with the Nextera XT Index Kit v2 Set A and B (Illumina, San Diego, U.S.A.) as described by the manufacturer. Quality controls of libraries were done using the Qubit 2.0 instrument (Thermo Fisher Scientific) and the 2100 Bioanalyzer instrument (Agilent Technologies, Santa Clara, U.S.A). Samples were diluted, pooled, spiked with an internal control (15% PhiX) and paired-end sequenced on the MiSeq Illumina platform using a flow cell with V2 chemistry (500 cycles). Negative controls were performed using pure water and elution buffer. In addition, microbial mock communities (Zymo Research, Irvine, California, USA) were run along as a standard and as a quality control for determining contamination bias from DNA extraction.

### Bioinformatic Microbiome Analysis Workflow

The bioinformatics analysis was performed as previously mentioned (Regier et al., 2018). Briefly, the paired end reads were joined and the primer sequences were removed. Reads with ambiguous base calls or with homopolymers longer than eight nucleotides were removed and duplicate sequences were merged and aligned against the SILVA-bases bacterial reference alignment (Quast et al., 2013). Using the Mothur implementation of the uchime algorithm, chimeric reads were removed, taxonomy was assigned and non-bacterial reads were removed. OTUs were created using Mothur and the taxonomy was reassigned to the ladder. In preparation for the analysis with Qiime, a phylogenetic tree and an OTU table in biom format was created. Subsequently, the alpha-diversity analysis and the taxa summary plots were created using the Qiime core diversity analysis script.

### Confirmation and Species Determination of *Bartonella* spp. by 16S-23S-rDNA-ITS-PCR and *rpoB*-PCR

*Bartonella* positive deer keds and corresponding muscle and full-blood samples, if available, were screened for *Bartonella* DNA. To detect *Bartonella* spp. specific DNA in deer keds, two PCRs were conducted: a 16S-23S-rDNA Internal Transcribed Spacer (ITS)- region-PCR (Cherry et al., 2009) and a PCR detecting the sequence for the *rpoB* gene (encoding the β-subunit of the bacterial RNA polymerase) was performed as previously described (Oksi et al., 2013). Both PCRs were conducted with the Platinum Taq Polymerase-Kit (Invitrogen, Schwerte, Germany). All PCR primers are listed in Table 1. Positive (*B. henselae* Houston ATCC 49882) and a negative (water) controls were always included. DNA was amplified in a Biometra T3000 thermocycler. Products were separated on an agarose gel, ethidium bromide stained and visualized under UV light. All PCR-products were sequenced (GATC, Konstanz, Germany). Sequences were analyzed using Chromas software (Technelysium, Version 2.6, South Brisbane, Australia) and compared to *Bartonella* spp. strains deposited in the NCBI databank using BLAST online tool to distinguish them on the species level.

**Table 1:** Targets, primers and amplicon sizes of the PCR-testing from deer keds, blood and muscels.

**Target sequence**	**Designation**	**Sequence (5^**′**^-3^**′**^)**	**Amplicon length**	**References**
*Bartonella* 16S-23S ITS region	325s	CTTCAGATGATGATCCCAAGCCTTCTGGCG	depending on *Bartonella* spp.	Cherry et al., 2009
	1100as	GAACCGACGACCCCCTGCTTGCAAAGCA	~500 bp
*Bartonella* spp.	prAPT0244	GATGTGCATCCTACGCATTATGG	406 bp	Oksi et al., 2013
*rpoB*	prAPT0245	AATGGTGCCTCAGCACGTATAAG	
*Acinetobacter baumanii*, carbapenemase *bla*_Oxa−51_	Oxa51-F Oxa51-R	TAATGCTTTGATCGGCCTTG TGGATTGCACTTCATCTTGG	353 bp	Woodford et al., 2006

### Nucleotide Sequence and Phylogenetic Analyses

Type alleles of the deer ked-derived partial *rpoB* fragments (Bs_GER_A GenBank: MH598359, Bs_GER_B GenBank: MH598360, and Bs_GER_C GenBank: MH598361) were used to search for similar sequences in nucleotide databases by Standard Nucleotide BLAST (BLASTN) at NCBI (https://www.ncbi.nlm.nih.gov) in June 2018. Search criteria were formatted with Expect min as 96 % and Expect max as 100 % to exclusively focus on close by entries within the ruminant lineage *Bartonella* species. Sorting was based on query coverage. BLASTN hits for all three *rpoB* type alleles were pooled into a single dataset, including removal of duplicate and triplicate entries. Phylogenetic analyses were performed using Molecular Evolutionary Genetics Analysis (MEGA) 6.06-mac (www.megasoftware.net/). To this end, the sequences were first aligned with ClustalW. Next, the partial *rpoB* fragments were either trimmed to a 406 bp fragment corresponding to a *B. henselae* Houston-1 *rpoB* fragment (AF171070) between nucleotide positions 1710 (TCGT…) and 2115 (…TCCA) or to a 285 bp fragment corresponding to a *B. henselae* Houston-1 *rpoB* fragment (AF171070) between nucleotide positions 1747 (ATTG….) and 2031 (…AGTA). The first *rpoB* sequence trimming corresponds to the maximum length fragment which can be obtained with *rpoB*-specific PCR primers prAPT0244 and prAPT0245 (Table 1). The second trimming corresponds to the maximum length fragment that is available for all the nucleotide database entries identified according to above BLASTN search criteria.

### Confirmation and Species Determination of *Acinetobacter* spp. by Oxa51-PCR

*Acinetobacter* spp. positive deer keds and corresponding muscle and full-blood samples, if available, were screened for *Acinetobacter* DNA. To detect *A. baumannii*-specific DNA, the gene for the *A. baumannii*-specific carbapenemase *bla*_Oxa−51_ was detected (Woodford et al., 2006), however, this chromosomally encoded carbapanemase does not contribute to carbapenem resistance of *A. baumanni* because it is not expressed. Primers are listed in Table 1. Positive (*A. baumannii*, patient isolate) and a negative (water) controls were always included.

## Results

### Sample Collection

**Figure 2:**
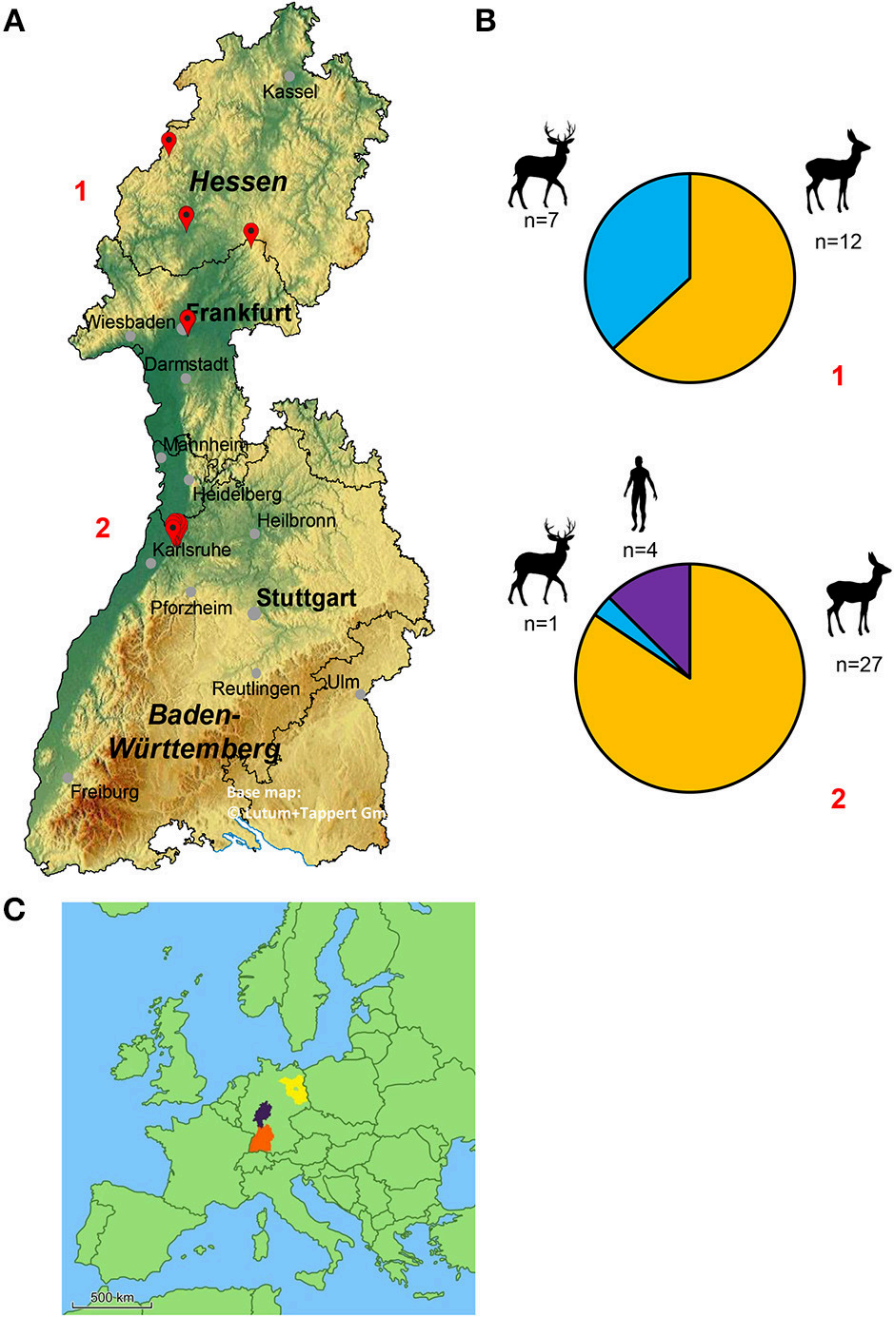
**(A)** Geographical map of the federal states of Hesse and Baden-Wuerttemberg (Germany) displaying the locations of deer ked collections. The red marks represent the location where deer keds were collected (from top to bottom, numbers in red: 1 = Hesse, 2 = Baden-Wuerttemberg). Four deer ked flies, which are not displayed, were collected in the greater area of Wittstock located in the federal state of Brandenburg (Germany). The base map was generated using EasyMap 11.0 ©Lutum+Tappert DV-Beratung GmbH. **(B)** Distribution of sampled deer keds and their hosts in relation to their location. 1: Hesse, 2: Baden-Wuerttemberg **(C)** Map of Europe. The exact locations of Hesse (purple), Baden-Wuerttemberg (orange) and Brandenburg (blue) are tagged.

130 deer keds were collected from 39 roe deer (*n* = 109), 8 fallow deer (*n* = 13) and 2 humans (*n* = 8). While the deer keds collected from animals had already shed their wings and a blood meal, the samples taken from humans still had their wings and did not start to feed on their hosts. Whenever possible, blood, or muscle samples were collected from the host animals. Full blood of 5 roe deer and muscle samples of 2 roe deer were taken. Locations of ked collections are given in Figure 2A,C. The number of sampled animals is summarized in Figure 2B.

### Next Generation Sequencing of Deer Keds for 16S rRNA Microbiome Analysis

To date, a broad and in detail microbiome investigation of whole deer keds has not been conducted. It has been reported previously that deer keds possibly also act as vectors for pathogenic bacteria (e.g., *B. schoenbuchensis*, (Vayssier-Taussat et al., 2016)). Hence, we were interested in identifying the microbial composition of deer keds sampled from different hosts throughout Germany.

In total, 130 deer ked samples were paired-end sequenced on the MiSeq Illumina platform, each resulting in a minimum sequencing depth of 5,000 reads. Sequences of samples with <5000 reads were excluded.

**Figure 3:**
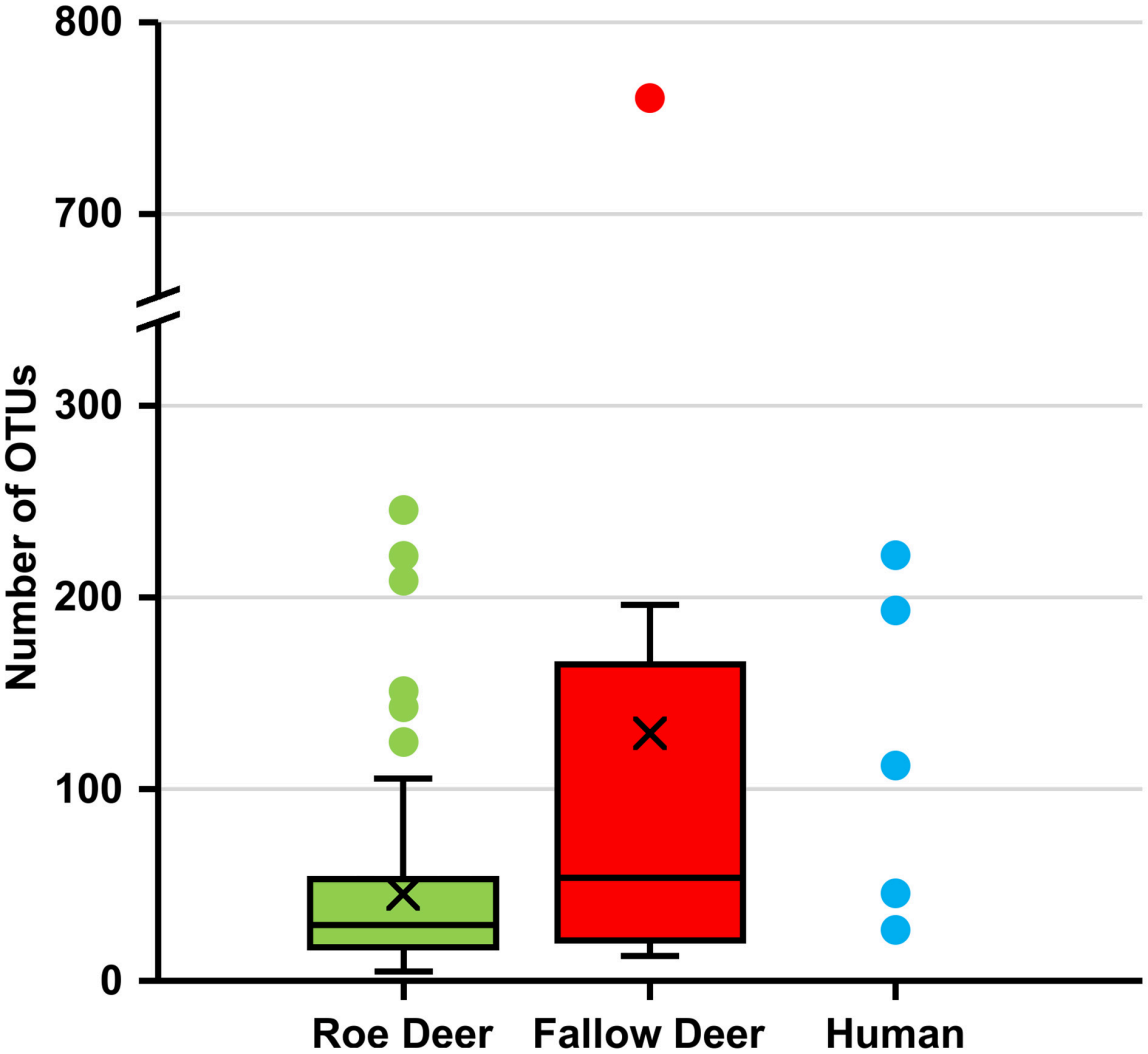
Number of OTUs in deer keds from roe deer, fallow deer and human. Number of operational taxonomic units (OTUs) at a sampling depth of 5,000 reads. Subsampling without replacement was repeated 1,000 times and averages reported.

The alpha diversity of deer keds sampled from humans and fallow deer reveals a higher number of OTUs compared to roe deer. This demonstrates higher species richness in deer keds sampled from humans and fallow deer (Figure 3).

**Figure 4:**
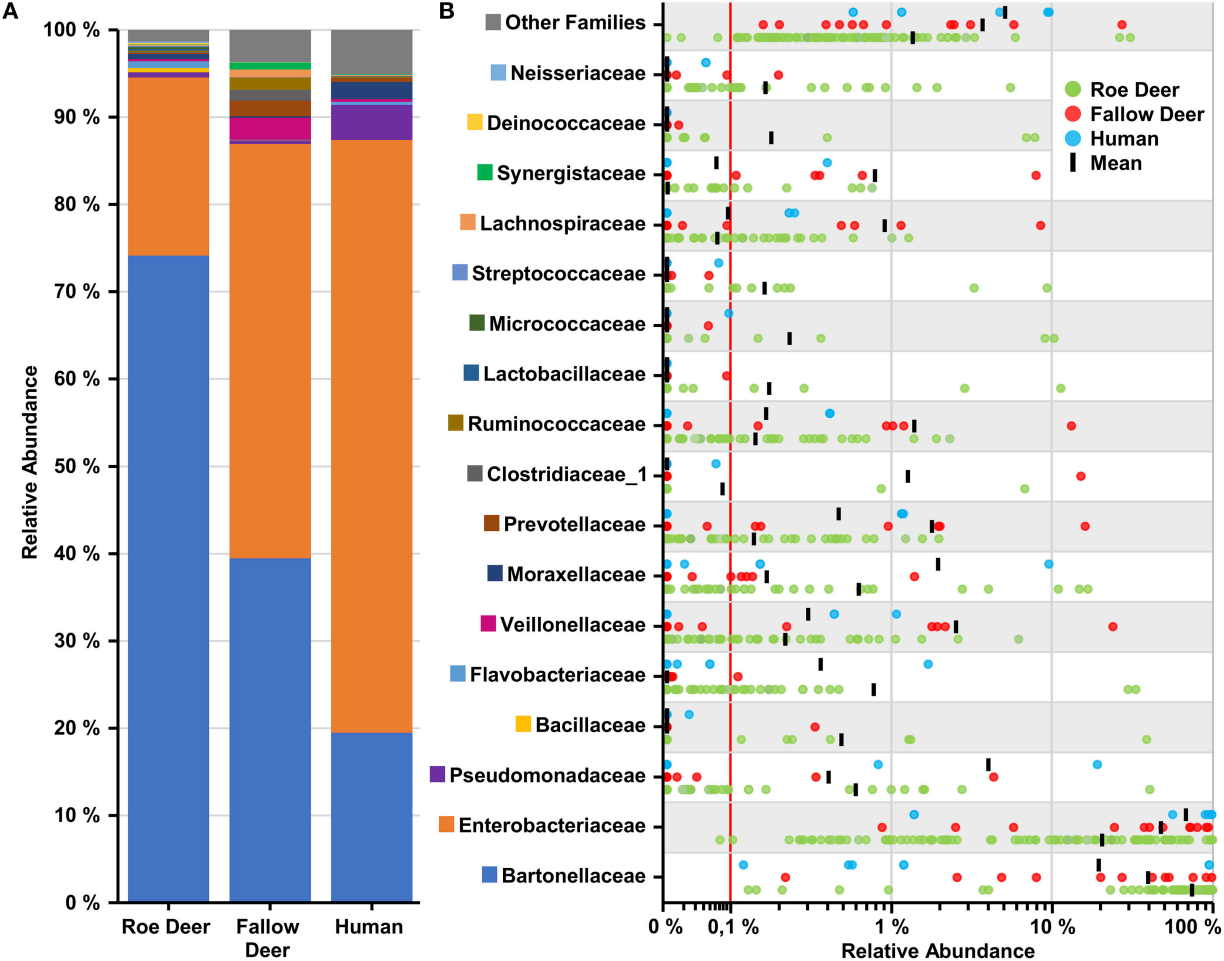
Overview of top 17 families found in deer keds. **(A)** Cumulative bar charts comparing relative family abundances for deer keds collected from row deer, fallow deer and humans. **(B)** Variation in relative abundance of each family in deer keds samples. Red line shows cutoff for noise. Families not in the top 17 by relative abundance are categorized as other families.

To examine the microbial taxonomic distribution of sampled deer keds, cumulative bar charts comparing relative family abundances were created. Comparing all three groups, we observed two dominant OTUs which are present, *Enterobacteriaceae* and *Bartonellaceae*. *Bartonellaceae* is found in a higher abundance in the roe deer group (~75%). The group of fallow deer (~40%) and humans (~20%) show a lower abundance of *Bartonellaceae*. The family of *Enterobacteriacea*, which was later identified as *Arsenophonus lipopteni* a known obligate intracellular symbiont of the deer ked (Nováková et al., 2016) was most dominantly abundant in the human group (~70%) followed by fallow deer (~45%) and roe deer (~20%) (Figure 4A). As seen in Figure 4B, which demonstrates the variation in relative abundances of the top 17 OTUs between the three groups, the group of fallow deer revealed a higher abundance of *Ruminococcaceae, Veillonellaceae*, and *Prevotellaceae*, which all are representatives of the oral or intestinal microbiome. Interestingly, *Acinetbacter* spp. was also in four deer ked samples, one which was identified as *A. baumannii*.

### Confirmation of Pathogen Detection by PCR, Sequencing, and Phylogenetic Analysis

#### Deer Keds

**Figure 5:**
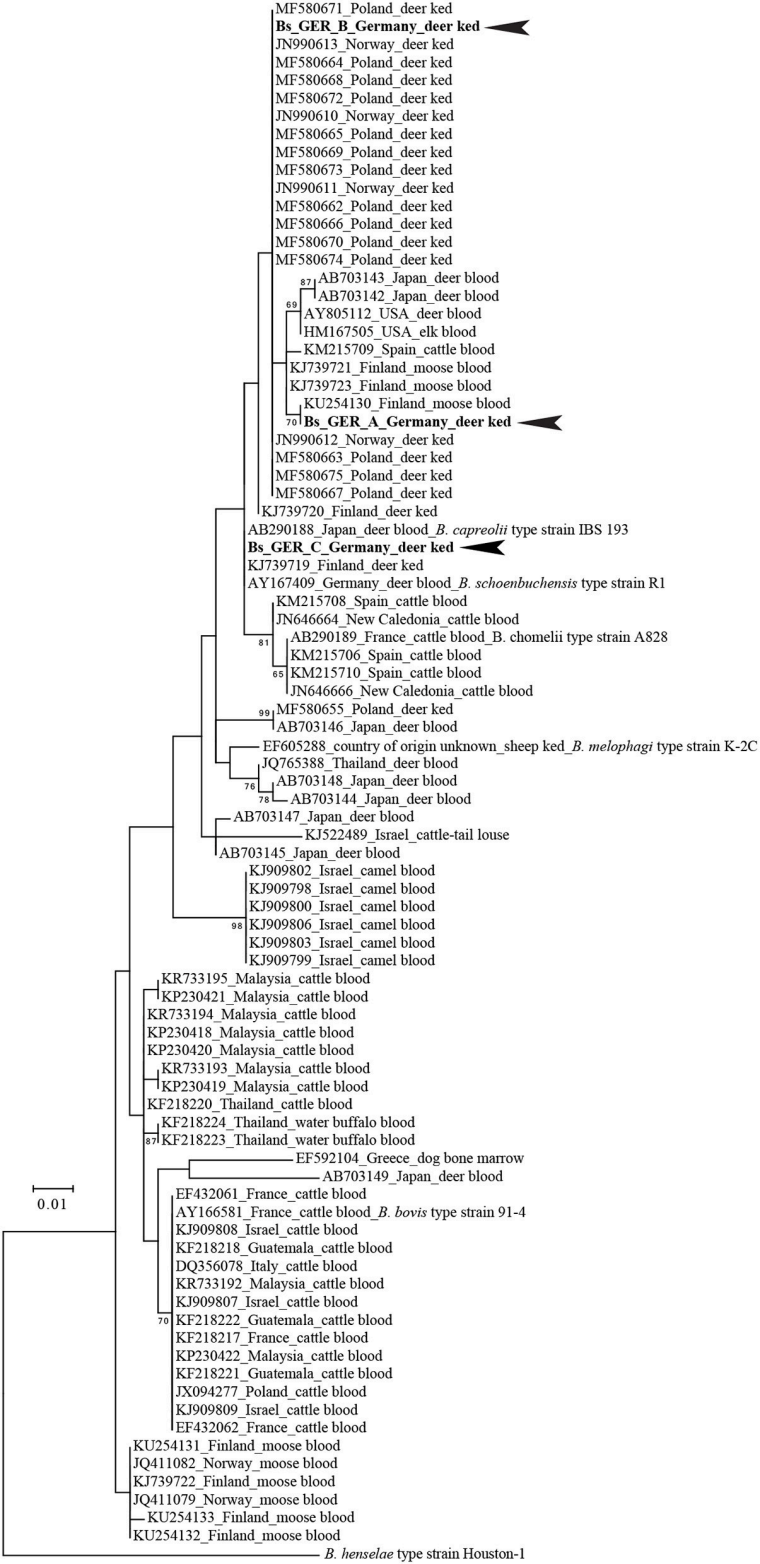
Phylogenetic positions of the type alleles of deer ked-derived partial *rpoB* fragments among their ≥96 % nucleotide identity BLASTN hits. Numbers on branches in the maximum likelihood tree indicate bootstrap support values derived from 500 tree replicas. Bootstrap values >60 are shown. Scale bar indicates nucleotide substitutions per site. *B. henselae* Houston-1 was used as outgroup for the *Bartonella* ruminant-lineage species. Identical phylogenetic positons were obtained whether the tree was constructed based on a 285 bp (shown) or a 406 bp *rpoB* fragment (see Materials and Methods).

Confirmatory PCRs on 95 deer ked samples revealed the presence of *B. schoenbuchensis* DNA in 93 samples. Three unique *rpoB*-alleles (Bs_GER_A GenBank: MH598359, Bs_GER_B GenBank: MH598360, Bs_GER_C GenkBank: MH598361) were detected. The *rpoB* allele “Bs_GER_A” was found in one deer ked from Karlsruhe, Germany, harvested from a fallow deer (*Dama dama*). The highest BLASTN sequence identity score (query coverage sorting) of the Bs_GER_A allele was 99.2 % (377/380 bp) with the *rpoB* fragments obtained from cattle blood samples in Spain (KM215709) or from elk blood samples in USA (HM167505). Bs_GER_A allele also had high BLASTN sequence identity scores to short *rpoB* fragments obtained from moose blood samples in Finland, e.g., 100% to KU254139. Phylogenetically the Bs_GER_A allele clustered with *rpoB* sequences retrieved from ruminant blood samples in Finland, Japan, Spain, and USA (Figure 5). The *rpoB* allele “Bs_GER_B” was detected in nine deer keds from Giessen, Germany and Frankfurt, Germany. These deer keds were either harvested from a fallow deer (*Dama dama; n* = 7) or from a roe deer (*Capreolus capreolus; n* = 1). The highest BLASTN sequence identity score (query coverage sorting) of the Bs_GER_B allele was 100 % (380/380 bp) with several *rpoB* fragments obtained from deer ked samples in Poland (e.g., MF580675). Phylogenetically, the Bs_GER_A allele also clustered strongly with short *rpoB* sequences retrieved from deer ked samples in Norway, e.g., JN990612 (Figure 5). The most common *rpoB* allele was “Bs_GER_C”, which was found in 83 deer ked samples and from all sampling sites. All of these deer keds were harvested from a roe deer (*Capreolus capreolus*). The highest BLASTN sequence identity score (query coverage sorting) of the Bs_GER_C allele was 100 % (387/387 bp) with the *rpoB* fragments obtained from a roe deer (*Capreolus capreolus*) blood *B. schoenbuchensis* type strain R1 isolate in Germany (AY167409) or from a human blood *B. schoenbuchensis* strain MVT06 isolate in France (HG977196). These similarities were also reflected in the phylogenetic clustering of the Bs_GER_C allele (Figure 5). No *Bartonella* spp. were detected in deer keds collected from humans. An *Acinetobacter* spp. OTU (operational taxonomic unit) was found in four deer ked samples. In one sample, the presence of *A. baumanii* DNA was confirmed by detecting the *A. baumannii* specific OXA 51 gene. Table 2 shows a summary of the allele distribution among all samples as well as the hosts and sampling sites.

**Table 2:** Distribution of *B. schoenbuchensis* rpoB-subtypes and prevalence in fallow deer and roe deer.

	**Sample**	**Sampling site (Germany)**	**Host**
Bs_GER_A	K7	Karlsruhe	1 fallow deer
Bs_Ger_B	G14	Giessen	1 fallow deer
	H1 (B,E,F)	Frankfurt	2 fallow deer, 1 roe deer (in one tube)
	H3	Frankfurt	1 fallow deer
	H4 (A,B)	Frankfurt	1 fallow deer
	H5	Frankfurt	1 fallow deer
	H6	Frankfurt	1 fallow deer
Bs_GER_C	W92	Forestry office Biedenkopf	1 roe deer
	W13	Vogelsberg	1 roe deer
	G3 (A,B)	Giessen	1 roe deer
	G5 (A,B)	Giessen	1 roe deer
	G6	Giessen	1 roe deer
	G7	Giessen	1 roe deer
	H1 (A)	Frankfurt	2 fallow deer, 1 roe deer (in one tube)
	H2 (B)	Frankfurt	1 roe deer
	H7 (A,B,D)	Frankfurt	1 roe deer
	H8	Frankfurt	1 roe deer
	K1 (B–M)	Karlsruhe	1 roe deer
	K2 (A–C)	Karlsruhe	1 roe deer
	K3 (A–C)	Karlsruhe	1 roe deer
	K4 (A–D, F)	Karlsruhe	1 roe deer
	K5 (A,B)	Karlsruhe	1 roe deer
	K6 (A–C)	Karlsruhe	1 roe deer
	K8 (A, C–H)	Karlsruhe	1 roe deer
	K9	Karlsruhe	1 roe deer
	K10	Karlsruhe	1 roe deer
	K11 (C, F–N, P–R)	Karlsruhe	1 roe deer
	K13	Karlsruhe	1 roe deer
	K14 (A,B)	Karlsruhe	1 roe deer
	K15 (A–C)	Karlsruhe	1 roe deer
	K17 (A)	Karlsruhe	1 roe deer
	K18 (A–B)	Karlsruhe	1 roe deer
	K19	Karlsruhe	1 roe deer
	K20	Karlsruhe	1 roe deer
	K22	Karlsruhe	1 roe deer
	K23	Karlsruhe	2 roe deer
	K24 (A,B)	Karlsruhe	1 roe deer
	K25 (A–C)	Karlsruhe	1 roe deer

#### Deer Blood

Blood from five roe deer (*Capreolus capreolus*) was available. The corresponding keds were *B. schoenbuchensis*-positive. DNA extracted from the blood was analyzed for the presence of *Bartonella* DNA by *rpoB*-specific PCR and Sanger-sequencing. One blood sample was positive with 100 % sequence identity with the Bs_GER_C rpoB allele. One sample showed an *rpoB* allele (GenBank: MH598362), which was different from deer ked “Bs_GER_A,” “Bs_GER_B,” and “Bs_GER_C” *rpoB* alleles (max ID). The highest BLASTN sequence identity score (query coverage sorting) of the unique deer blood allele was 100% (406/406 bp) with the *rpoB* fragment of the *B. capreoli* type strain IBS193. Therefore, this blood sample was positive for *B. capreoli*, not for *B. schoenbuchensis*. Three blood samples remained negative. *A. baumannii* was not detected in any of the blood samples, analyzed with OXA 51 gene-specific PCR.

#### Deer Muscle

Muscle samples from two roe deer (*Capreolus capreolus*), whose keds were *B. schoenbuchensis*-positive, were also analyzed for the presence of *Bartonella* DNA by *rpoB*-specific PCR and Sanger-sequencing. One sample remained negative while the other sample was positive for *B. schoenbuchensis* DNA with 100 % identity to the most prevalent *rpoB* allele “Bs_GER_C” detected in the deer keds.

## Discussion

Blood-sucking arthropods are vectors for many human and animal pathogenic bacteria. Whereas a plethora of data regarding transmission of pathogens to animals and humans by ticks and fleas is available (Regier et al., 2016), virtually nothing is known about pathogen transmission by deer keds. Based on our recent experiences in performing tick metagenomics (Regier et al., 2018), where we identified in total six potentially pathogenic genera being present within the ticks, we expected a broad variety of human or animal pathogenic bacteria in these deer keds. Furthermore, *Borrelia burgdorferi* DNA and *Anaplasma phagocytophilum* DNA has already been detected in deer keds in earlier studies (Buss et al., 2016). However, we found a surprisingly low diversity of bacteria within these insects suggesting that deer keds are not transmitting a broad spectrum of pathogens. The deer ked microbiome mainly consisted of two OTUs: *Arsenophonus* spp. and *Bartonella* spp. *Arsenophonus lipopteni* is a well-known endosymbiont in deer keds with unknown biological function (Nováková et al., 2016). *B. schoenbuchensis* DNA was previously found in moose, roe deer, red deer, and cattle so probably these ruminants represent the reservoir hosts for these species (Chang et al., 2000; Bermond et al., 2002; Rolain et al., 2003; Maillard et al., 2004; Adamska, 2008; Duodu et al., 2013; Welc-Faleciak et al., 2013; Korhonen et al., 2015). *Lipoptena cervi* is suspected to act as the main vector for *B. schoenbuchensis*, it was found by cultivation and via molecular methods in adult *L. cervi* (Dehio et al., 2004; Matsumoto et al., 2008; Duodu et al., 2013; Bruin et al., 2015; Korhonen et al., 2015; Szewczyk et al., 2017), *L. mazamae* (Reeves et al., 2006) and feeding ticks (Matsumoto et al., 2008). Furthermore, *B. schoenbuchensis* was shown to colonize the midgut of deer keds (Dehio et al., 2004). Replication in the arthropod is a crucial prerequisite for deer keds to act as a natural reservoir host or a natural vector. Several studies showed the presence of *Bartonella* DNA in deer ked pupae (Duodu et al., 2013), in pupae and adult winged deer keds (Korhonen et al., 2015) and of *B. schoenbuchensis* DNA in winged and wingless deer keds and in larvae (Bruin et al., 2015). Also, no *Bartonella* spp. were cultured from moose with no deer ked infestation (Duodu et al., 2013). All these findings make it very likely that *L. cervi* is the vector for *B. schoenbuchensis* and that the bacteria can be transmitted transstadially. One deer ked sample was positive for *B. capreoli* but the corresponding deer ked was not. This could lead to the conclusion, that *B. capreoli* is not transmitted by deer keds. Epidemiologically, our data suggest that *B. schoenbuchensis* alleles derived from fallow deer and roe differ as the *rpoB* allele Bs_GER_B is nearly exclusively seen in fallow deer and Bs_GER_C in roe deer. Reasons for this fact remain speculative but host specificity is common among *Bartonella* spp. (Dehio, 2005; Mändle et al., 2005; Maggi et al., 2011) and this finding might demonstrate the process of host adaption of *B. schoenbuchensis* by so far unknown molecular mechanisms. It might be speculated that fallow deer had to less time to adapt to *B. schoenbuchensis* Bs_GER_C and roe deer to *B. schoenbuchensis* Bs_GER_B, respectively.

Although no *Bartonella* spp. were detected in deer keds collected from humans, there is a possibility for humans to be infested with these bacteria since *Bartonella* spp. were detected in a huge number of ked samples. To date, it is unclear if and which diseases can be caused by *B. schoenbuchensis* in humans but it is suspected to cause the so called “deer ked dermatitis.” Persistent, therapy resistant, pruritic papules can form one to 24 h after deer ked contact and it was shown that immunologic mechanisms are probably involved in the pathogenesis (Rantanen et al., 1982). Dehio et al. (2004) proposed *B. schoenbuchensis* as a cause of the dermatitis because of the similarity to the primary manifestation of cat scratch disease caused by *B. henselae* but clear evidence for this is still missing. Other ruminant-associated *Bartonella* spp. have also been shown to cause diseases in humans and animals. The role of *B. bovis* as the causative agent of endocarditis in cows has already been reported twice (Maillard et al., 2007) but a *B. bovis* bacteremia had no effect on milk production or reproduction in cattle (Maillard et al., 2006). *B. melophagi* was isolated from the blood of two sick women suffering from fatique, muscle weakness, muscle pain and fever, but its role in the pathogenesis of these symptoms remains unclear (Maggi et al., 2009). In another study, *B. schoenbuchensis* was isolated from the blood of a patient again suffering from fatigue, muscle pain and fever. The patient had a history of tick bites and was seronegative for Lyme borreliosis (Vayssier-Taussat et al., 2016). To analyze whether deer ked transmitted *B. schoenbuchensis* might contribute to the pathogenesis of deer ked dermatitis or to those unspecific entities attributed to ruminant-associated *Bartonella* spp., it would be necessary to perform, e.g., studies in which the presence of anti-*B. schoenbuchensis*-antibodies in deer ked-exposed patients would be systematically analyzed. However, serological tools to perform such surveys are not available and, moreover, no cut-off values for *B. schoenbuchensis* serology have been determined.

In 2017, the World Health Organization listed *A. baumannii* as one of the top pathogens for which new antibiotics are urgently needed (WHO., 2017). It is known that *A. baumannii* is present in livestock (e.g., chicken and geese) and in wild storks (Wilharm et al., 2017). *Acinetobacter* DNA was also found in ectoparasites of domestic animals (Kumsa et al., 2012). Moreover, *A. baumannii* DNA is present in up to 21% of body lice (La Scola and Raoult, 2004). Therefore, transmission of this emerging pathogen might be promoted by various arthropod species. Although we detected non-baumannii *Acinetobacter* spp. in only three specimens and *A. baumannii* only once in a deer ked, it can nevertheless be discussed that deer keds contribute to the distribution of *A. baumannii* in animals and humans. In conclusion, *L. cervi* should be considered as a highly likely vector for *B. schoenbuchensis* and a potential vector for *Acinetobacter* spp. Because of the fact that symptoms attributed to *B. schoenbuchensis* infections are of limited scientific evidence, it might be worth in future to systematically analyze whether *B. schoenbuchensis* transmitted by deer keds contributes infectious diseases in animals and humans.

## Data Availability Statement

Microbiome sequencing data have been submitted to the NCBI Short Read Archive repository under the SRA accession number SRP156522 (https://www.ncbi.nlm.nih.gov/sra/SRP156522). The various *rpoB* alleles were deposited in GenBank under the accession numbers MH598359 (Bs_GER_A), MH598360 (Bs_GER_B, and MH598361 (Bs_GER_C).

## Author Contributions

VK performed the experimental design. YR performed sample collection, DNA extraction and PCRs. KK, MW, and TH performed next generation sequencing and bioinformatic analysis. AP performed phylogenetic analysis. YR, KK, MW, AP, SG, TH, and VK performed writing of the manuscript. All authors read and approved the final manuscript.

### Conflict of Interest Statement

The authors declare that the research was conducted in the absence of any commercial or financial relationships that could be construed as a potential conflict of interest.

## Abbreviations

A: Acinetobacter
B: Bartonella
L: Lipoptena
OUT: Operational Taxonomic Unit.
